# The Accuracy of the CBCT‐Based 3‐Dimensional Replica of the Donor Tooth in Autotransplantation

**DOI:** 10.1002/cre2.70032

**Published:** 2024-10-29

**Authors:** Jessica Juslin, Tuija Teerijoki‐Oksa, Päivi Jääsaari, Marja Ekholm, Pekka Vallittu, Lippo Lassila, Hanna Thorén

**Affiliations:** ^1^ Department of Oral and Maxillofacial Surgery University of Turku Turku Finland; ^2^ Department of Oral and Maxillofacial Diseases Turku University Hospital Turku Finland; ^3^ Department of Pediatric Dentistry and Orthodontics University of Turku Turku Finland; ^4^ Department of Oral Pathology and Oral Radiology University of Turku Turku Finland; ^5^ South West Finland Imaging Centre Turku University Hospital Turku Finland; ^6^ Department of Biomaterials Science University of Turku Turku Finland; ^7^ City of Turku, Welfare Division Turku Finland

**Keywords:** 3D replica, replica tooth, tooth transplantation

## Abstract

**Background:**

This study evaluated the accuracy of the CBCT reconstruction model compared to the natural tooth and the accuracy of the replica tooth compared to the natural tooth.

**Objective:**

The hypothesis was that a replica tooth could be used as a surgical guide in autotransplantation.

**Methods:**

Three teeth were chosen and a CBCT reconstruction model was formed from each tooth. STL‐data was transferred to a milling machine and replica teeth were milled from PEEK. A digitized surface model was prepared from the natural and the replica teeth by a stereophotogrammetry scanner. The surface model from the optical scan of the natural tooth was compared to the CBCT reconstruction model and the surface model of the replica tooth. The models were matched on each other, and surface‐based rigid registration was performed between the surface models. Distances were calculated and visualized by MATLAB.

**Results:**

The CBCT reconstruction model and the natural tooth were compared. The largest euclidean distance was found at the root tip in the premolar (0.93 mm) and at the furcation area in the molar (2.3 mm). When the natural tooth and the replica tooth were compared, the largest euclidean distance was found at the root tip in the premolar (1.5 mm) and at the furcation area in the molar (1.9 mm).

**Conclusion:**

A CBCT scan maintains sufficient image quality for tooth autotransplantation planning. The replica tooth corresponded in size and shape to the natural tooth in terms of clinically expected need of precision.

## 1 Background

In tooth transplantation, a donor tooth is surgically removed and positioned in a recipient area of the same individual. The process of healing in a transplanted tooth is similar to a replanted avulsed tooth. The transplanted tooth can survive without endodontic treatment if the vascular and nerve bundles in the apical area and the periodontal ligament recover after surgery (Paulsen, Andreasen, and Schwartz 1995; Machado et al. 2016). Therefore, delicate handling of transplant is required to maintain the health of the periodontal ligament. Moreover, the root surface should not be allowed to dry (Blomlöf 1981; Andreasen and Kristerson 1981) as drying initiates osteogenesis in the alveolar bone, which further predisposes for ankylosis (Andreasen and Kristerson 1981). Investigators of recently reported meta‐analyses and umbrella reviews have shown that the overall success and survival of transplanted teeth, in general, has been high in teeth with an open apex (Atala‐Acevedo et al. 2017; Kim, Choi, and Pang 2019; Tan et al. 2023; Jaber et al. 2024).

Digital planning and rapid prototyping have gained a lot of attention during the past decade in diagnosis, treatment planning, and dentoalveolar surgery. Manufacturing a 3‐dimensional replica by rapid prototyping could potentially help surgeons prepare the recipient site to match to the contour of the donor tooth, thus minimizing manipulation of the donor tooth. When planning tooth transplantation, a cone beam computed tomography (CBCT) scan is taken as the possible donor tooth and the recipient site (Kim, Choi, and Pang 2019). A stereolithographic (STL) model could be segmented from the CBCT data and a 3D model of the tooth to be transplanted could be fabricated by using CAM methodologies milling or 3D printing. Several investigators have previously examined different techniques of utilizing 3D technology in tooth transplantation (Kim, Choi, and Pang 2019; Zhang, Han, and Zhong 2024; Shahbazian et al. 2010; Verweij et al. 2020).

The purpose of this study was to determine the accuracy of the CBCT reconstruction model compared to the optical scan of the natural donor tooth and to determine the accuracy of the 3‐dimensional replica tooth compared to the natural tooth.

The hypothesis of this study was that a 3‐dimensional replica tooth could be used as a surgical guide in tooth transplantation at the clinical setting, as the replica tooth is accurate when compared to the corresponding natural tooth.

## 2 Methods

### 2.1 Study Sample

Three teeth of unknown donors were collected from the Institute of Dentistry, University of Turku, Finland. One premolar, one canine, and one molar were chosen as model teeth to represent the most frequently autotransplanted teeth (Juslin et al. 2020).

### 2.2 Ethical Aspects

According to Finnish legislation, tissue samples that have been taken for therapeutic purposes may be donated and used for medical research, if no personal data is used and if permission is granted from the unit from whose activities the sample was taken (Act on the Medical Use of Human Organs). The three teeth that were used in the present study were collected from the Institute of Dentistry, University of Turku, Finland. The donors of the teeth are unknown. No personal data was registered when the teeth were donated. Permission to use the teeth for the present study was granted by the Institute of Dentistry. Approval from the ethics committee or informed consent from the patients is therefore not required.

### 2.3 Imaging Protocol

The teeth were positioned in putty impression material (polyvinyl siloxane) and CBCT‐scanning was performed using Planmeca Promax 3D–scanner (Planmeca, Finland) with voxel size 200 μm, tube voltage 90 kV, current 6.3 mA, and exposure time of 12 s.

### 2.4 Data Processing

The CBCT scans in DICOM format were opened in MATLAB (Mathworks, Natick, MA, R2018b) and the 3D volume rendered teeth were segmented and triangulated into STL‐format (CBCT reconstruction model). First all voxels over 1600 HU were chosen and a surface graph was formed by using the Marching Cubes algorithm. The root canals were filled with a separate algorithm. The segmentation model was formed by using the isosurface function in MATLAB.

### 2.5 Replica Teeth Manufacturing

The digitized information in the STL format was transferred to a 3D milling machine, where the replica teeth were milled. The replicas were milled from a block of polyaryletheretherketone (PEEK) in a M5 milling unit (Zirkonzahn, Italy). The M5 milling unit uses 5 + 1 milling technology.

### 2.6 Digitizing Natural Teeth and Replica Teeth

A digitized surface model was prepared from the natural teeth and the replica teeth by a stereophotogrammetry scanner Atos Core (GOM, Germany). Each tooth was scanned in two steps: first the crown area was scanned and then the root area. After this, the registered half‐models were combined forming a model of the whole tooth.

### 2.7 Accuracy Assessment of CBCT

The surface model from the optical scan of the natural tooth was compared to the CBCT reconstruction model of the same tooth. The models were matched on each other, and a surface‐based rigid registration was performed between the surface models.

Euclidean and normal distances were calculated and visualized by MATLAB (Mathworks, Natick, MA, R2018b). The Euclidean distance measures the distance to the closest point on the other surface. The normal distance measures the directed distance to the corresponding point on the other shape. The software output provided the minimum, the maximum, mean, and median values from each distance distribution. The computed distances were also expressed on the CBCT reconstruction model using a blue‐to‐yellow color mapping.

### 2.8 Accuracy Assessment of Replica Teeth

The surface model from the optical scan of the natural tooth was compared to the surface model of the replica teeth. The models were matched on each other, and surface‐based rigid registration was performed between the surface models.

Euclidean and normal distances were calculated and visualized by MATLAB (Mathworks, Natick, MA, R2018b). The software output provided the minimum, the maximum, mean, and median values from each distance distribution. The computed distances were also expressed on the scan of the natural tooth using a blue‐to‐yellow color mapping for semiquantitative visual analysis.

## 3 Results

### 3.1 The Accuracy of the CBCT Reconstruction Model Compared to the Optical Scan of the Natural Tooth

**Figure 1:**
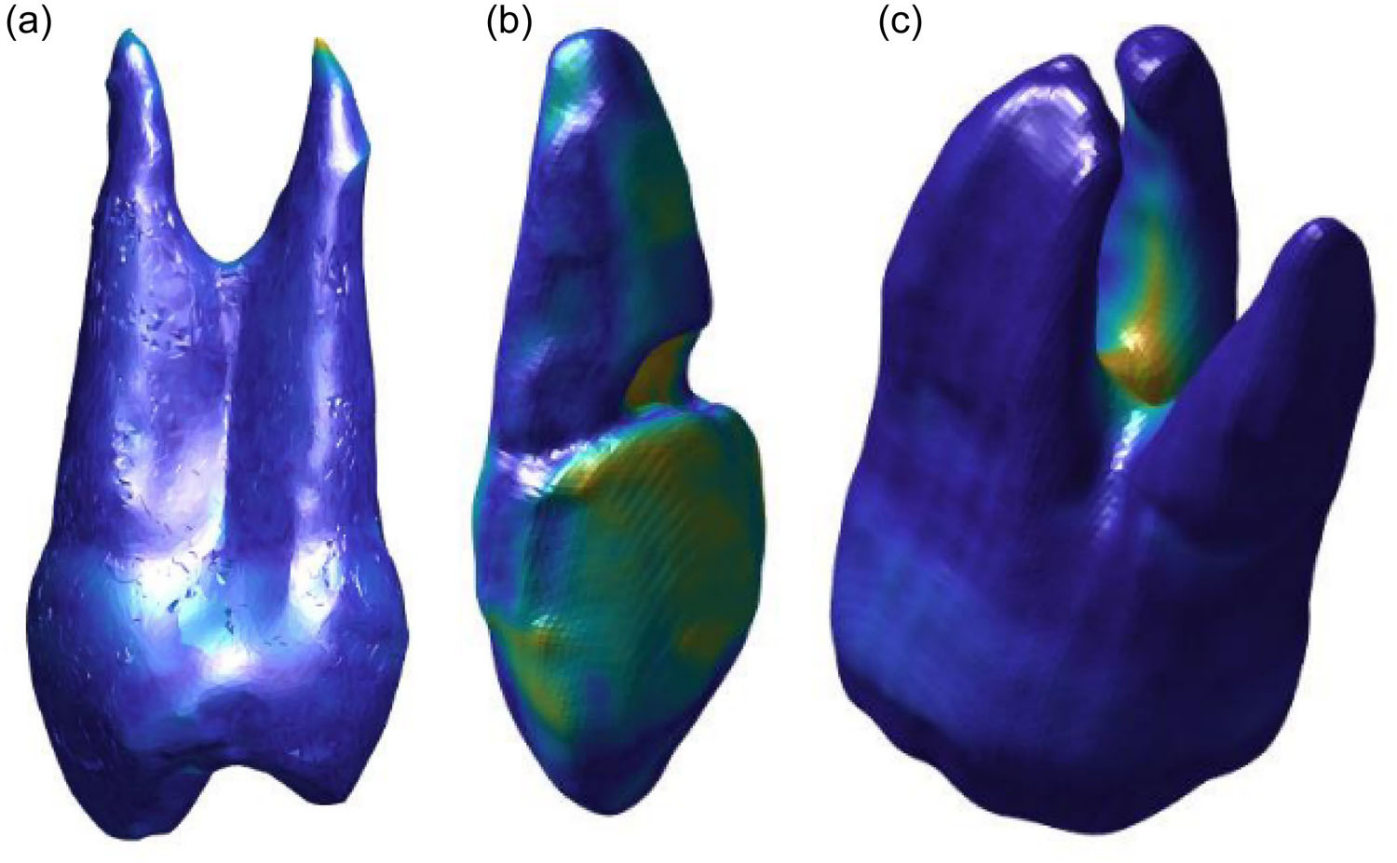
Computed Euclidean distances between the surface models of the optical scan of the natural teeth and the CBCT reconstruction models in blue‐to‐yellow color mapping (a) of the premolar, (b) of the canine, and (c) of the molar tooth. Dark blue represents the minimum Euclidean distance and light yellow the maximum Euclidean distance.

**Figure 2:**
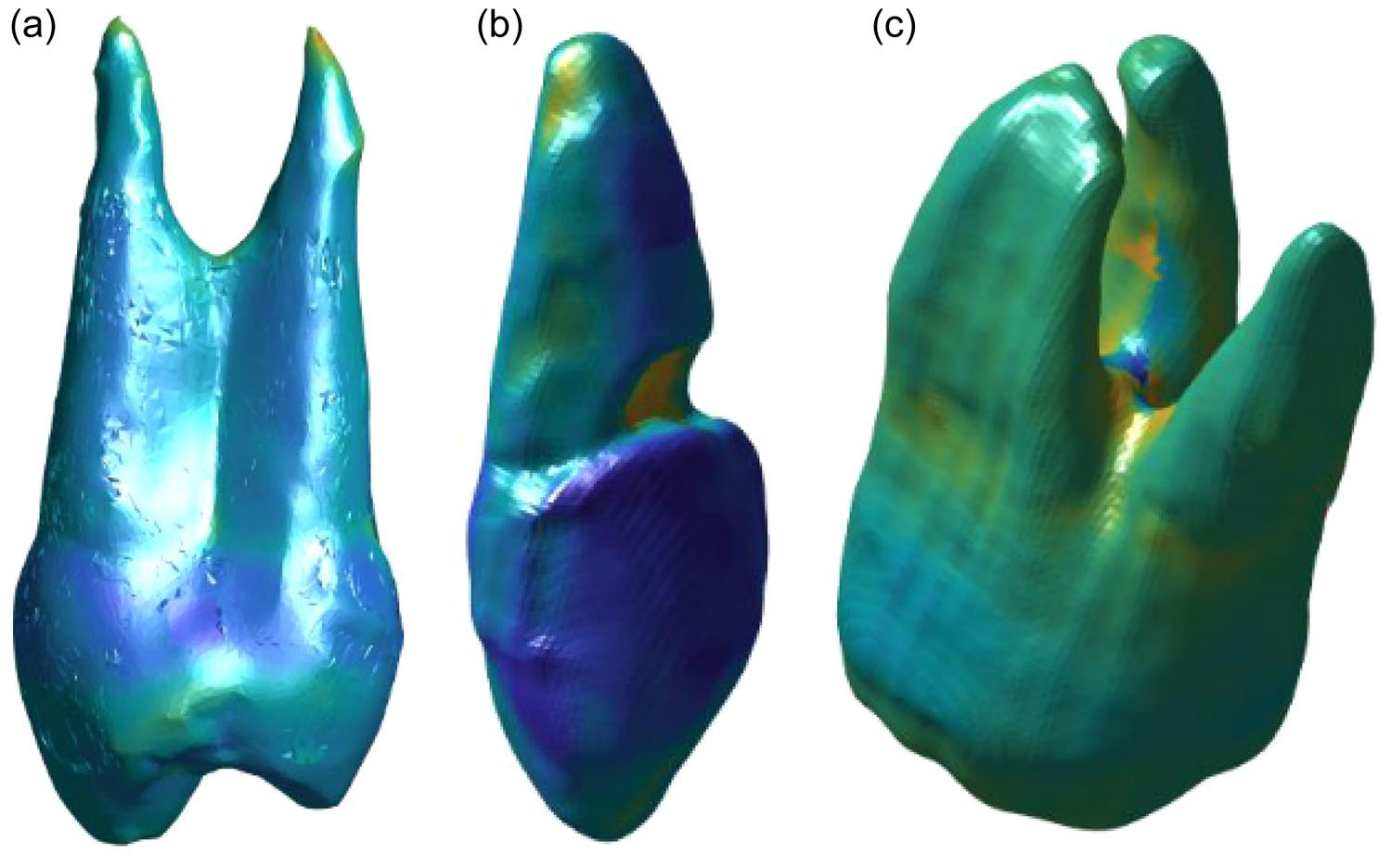
Computed normal distances between the surface models of the optical scan of the natural teeth and the CBCT reconstruction models in blue‐to‐yellow color mapping (a) of the premolar, (b) of the canine, and (c) of the molar tooth. In the case of bright yellow, the natural tooth was bigger, and in the areas of dark blue smaller than the CBCT reconstruction model.

Computed distances between the surface models of the optical scan of natural teeth and the CBCT reconstruction models are presented in Table 1 and in Figures 1a–c and 2a–c.

**Table 1:** Computed distances between the surface models of the optical scan of the natural teeth and the CBCT reconstruction models.

Tooth	Euclidean distances		Median	Max
	Min	Mean		
Premolar	0.00300	0.07	0.0917	0.928
Canine	0.00424	0.199	0.154	1.07
Molar	0.00285	0.208	0.126	2.34

	Normal distances			
Tooth	Min	Mean	Median	Max
Premolar	−0.461	−0.0448	−0.0368	0.282
Canine	−0.563	−0.0877	−0.0791	1.04
Molar	−1.69	−0.0217	−0.0326	1.56

	Normal distances, absolute			
Tooth	Min	Mean	Median	Max
Premolar	0.00000516	0.0704	0.0522	0.461
Canine	0.00000700	0.164	0.124	1.04
Molar	0.00000200	0.130	0.0941	1.69

In the blue‐to‐yellow color mapping (see Figure 1a–c), dark blue represented the minimum Euclidean distance, and bright yellow the maximum Euclidean distance. The largest distance was found at the root tip in the premolar (0.93 mm), at the area of the chipped filling in the canine (1.1 mm) and at the root furcation area in the molar (2.3 mm). The mean ranged from 0.11 to 0.21 mm and the median ranged from 0.092 to 0.15 mm.

The normal distances were expressed by the blue‐to‐yellow color mapping (see Figure 2a–c). In this case, dark blue represented most negative values and bright yellow most positive values. Negative values indicated that the original tooth is smaller than the CBCT reconstruction model. Positive values indicated that the natural tooth is larger than the CBCT reconstruction model. The largest deviation was found at the root tip in the premolar (−0.46 mm) at the area of the chipped filling in the canine (1.0 mm) and at the root furcation area in the molar (−1.7 mm). The absolute mean ranged from 0.07 mm to 0.16 mm and absolute median ranged from 0.05 to 0.12 mm.

### 3.2 The Accuracy of the Replica Tooth Compared to the Natural Tooth

**Figure 3:**
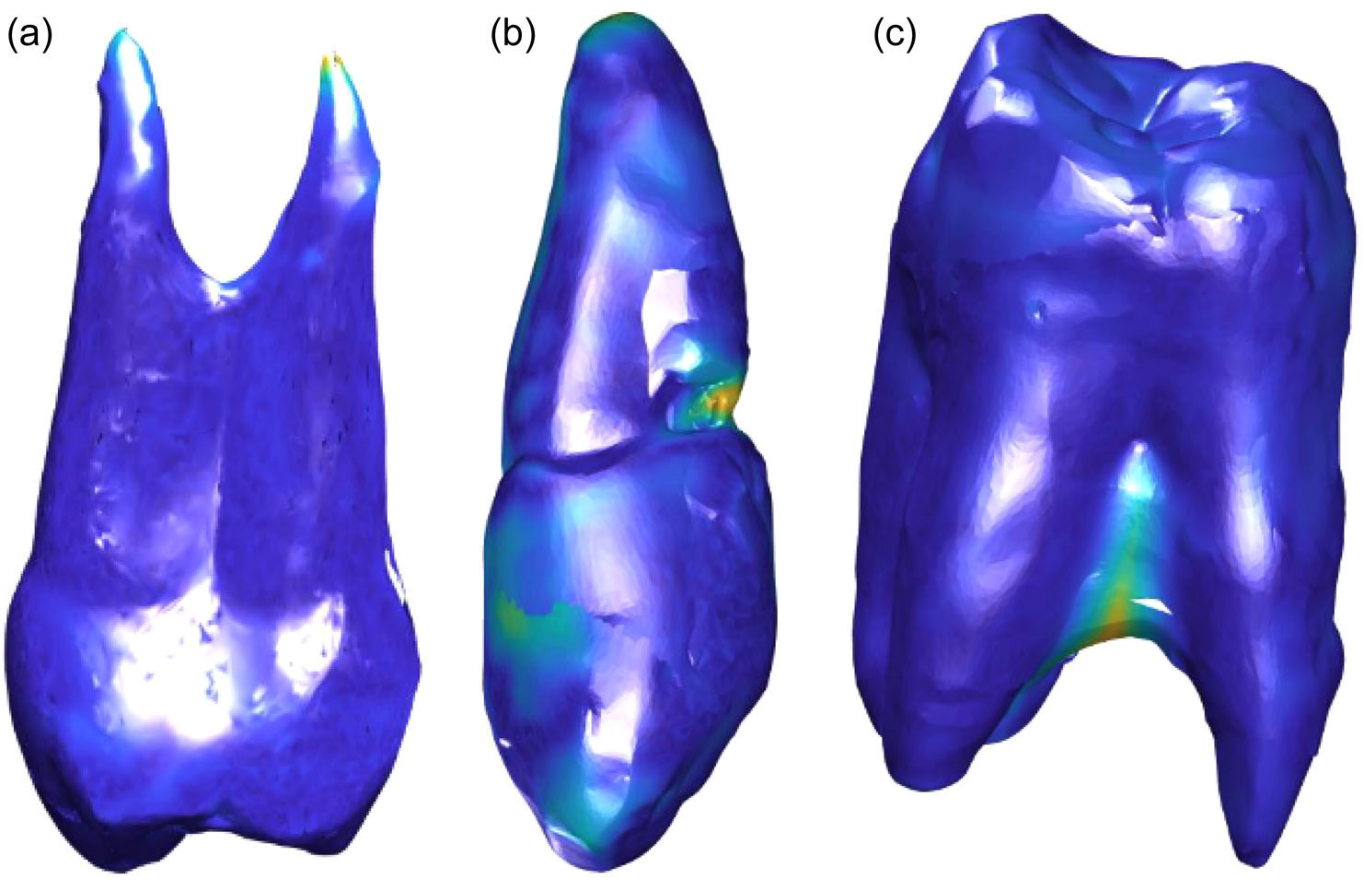
Computed Euclidean distances between the surface models of the optical scan of the natural teeth and the replica teeth in blue‐to‐yellow color mapping (a) of the premolar, (b) of the canine, and (c) of the molar tooth. Dark blue represents the minimum Euclidean distance and light yellow the maximum Euclidean distance.

**Figure 4:**
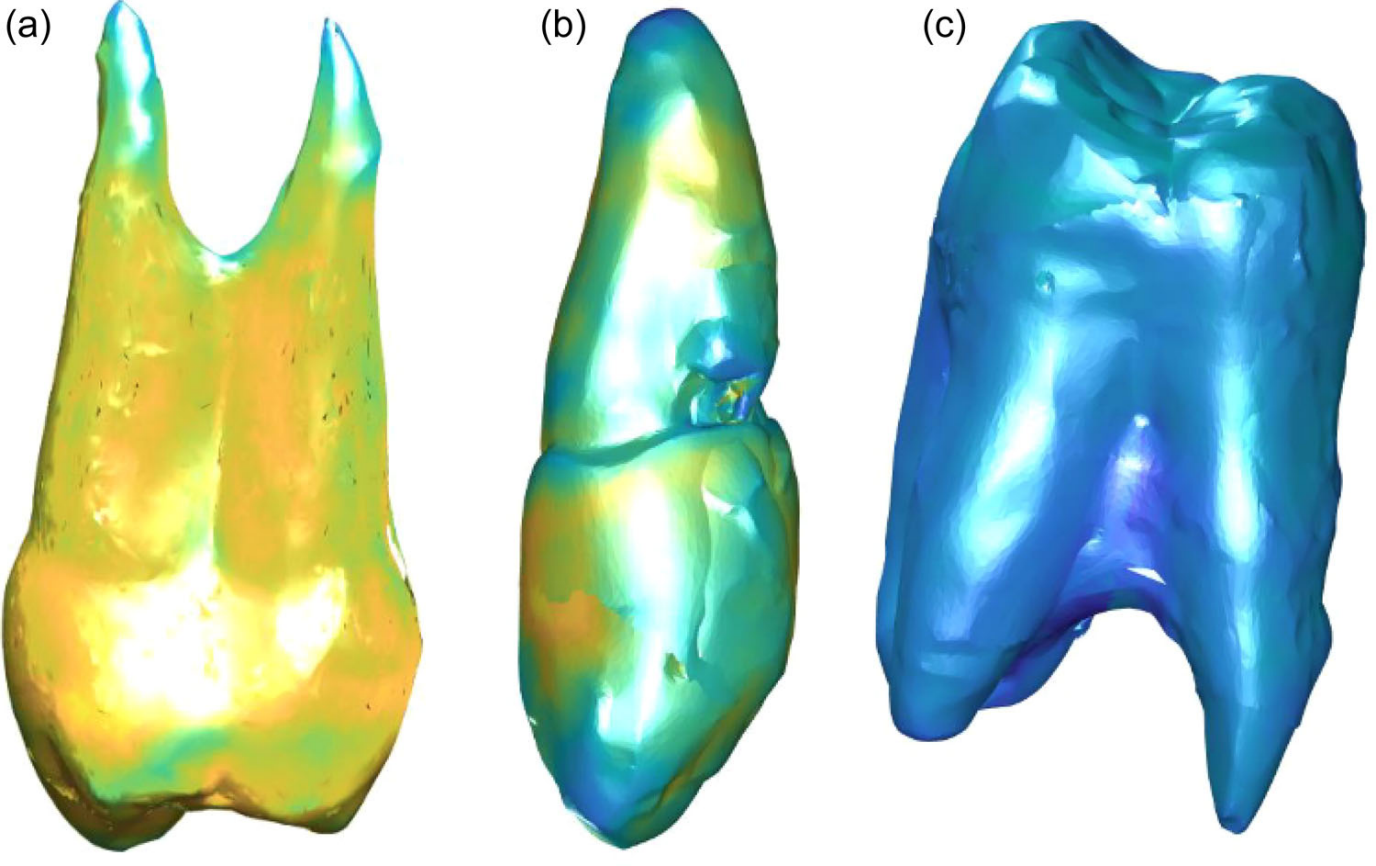
Computed normal distances between the surface models of the optical scan of the natural teeth and the replica teeth in blue‐to‐yellow color mapping (a) of the premolar, (b) of the canine, and (c) of the molar tooth. In the case of bright yellow, the natural tooth was bigger and in the areas of dark blue smaller than the replica tooth.

Computed distances between the surface models of the optical scan of the natural teeth and the replica teeth are presented in Table 2 and in Figures 3a–c and 4a–c.

**Table 2:** Computed distances between the surface models of the optical scan of the natural teeth and the replica teeth.

Tooth	Eucleudian distances			
	Min	Mean	Median	Max
Premolar	0.00490	0.112	0.0913	1.49
Canine	0.00700	0.199	0.149	1.13
Molar	0.00300	0.183	0.126	1.94

	Normal distances			
Tooth	Min	Mean	Median	Max
Premolar	−1.03	0.0121	0.0146	0.345
Canine	−0.92	0.0155	0.000630	0.817
Molar	−0.934	−0.0116	−0.00489	0.814

	Normal distances, absolute			
Tooth	Min	Mean	Median	Max
Premolar	0.0000106	0.0761	0.0511	1.03
Canine	0.0000132	0.153	0.117	0.920
Molar	0.00000800	0.124	0.0904	0.934

In the blue‐to‐yellow color mapping (see Figure 3a–c) dark blue represented the minimum Euclidean distance and bright yellow the maximum Euclidean distance. The largest distance was found at the root tip in the premolar (1.5 mm), at the area of the chipped filling in the canine (1.1 mm) and at the root furcation area in the molar (1.9 mm). The mean ranged from 0.11 to 0.20 mm and the median ranged from 0.091 to 0.15 mm.

The normal distances were expressed by the blue‐to‐yellow color mapping (see Figure 4a–c). In this case, dark blue represented most negative values and bright yellow most positive values. Negative values suggest that the natural tooth is smaller than the replica tooth. Positive values suggest that the natural tooth is larger than the replica tooth. The largest deviation was found at the root tip in the premolar (−1.0 mm) at the area of the chipped filling in the canine (−0.92 mm) and at the root furcation area in the molar (−0.93 mm). The absolute mean ranged from 0.076 to 0.15 mm and absolute median ranged from 0.051 to 0.12 mm.

## 4 Discussion

The aim of the present study was to determine the accuracy of the dimensions of the CBCT reconstruction model of the natural tooth compared to the optically scanned and CAM‐fabricated counterpart. This study was undertaken to confirm dimensional precision and equivalency of the natural tooth and the replica tooth for feasibility analysis of the method prior clinical evaluation. It was found that the absolute mean was ≤ 0.16 mm and absolute median ≤ 0.12 mm when we compared the CBCT reconstruction model to the optical scan of the corresponding natural donor tooth. The most problematic area to reproduce by scanning was the furcation area of the molar where the maximum absolute normal distance was 1.7 mm. The Euclidean distance probably included some in‐plane inaccuracy due to non‐aligned vertices between the meshes. The normal distance mostly ignores this tangential error that might occur when the surface models were matched on each other.

The mean absolute deviation in normal direction between the original tooth and replica tooth ranged from 0.076 to 0.15 mm. The most problematic area to reproduce by scanning and milling was the furcation area of the molar where the maximum absolute normal distance was 1.9 mm. The maximum absolute distance ranged from 0.93 to 1.0 mm. Shahbazian et al. (2010) CBCT scanned a dry dentate mandible and compared the segmented tooth to the 3D model of the tooth obtained by optical scanning. In 79% of the surface, the deviation was ≤ 0.25 mm. The largest deviations up to 2.5 mm occurred on the root surfaces that were in close contact with the cortical bone. In the present study, the effect of adjacent bone to the scanning and reproduction precision was not considered but it needs to be considered when the concept is clinically evaluated.

If the transplanted tooth has multiple roots, the surgeon usually prepares a cavity to the alveolar bone according to the outermost surface contour of the roots rather than preparing separate cavities of each root. Therefore, a deviation less than 2.0 mm in the furcation area probably does not matter when this concept is tested in the clinical setting. Regarding the surgical procedure, in particular, delicate handling of the transplant is required to maintain the health of the periodontal ligament. The donor tooth is supposed to be fitted atraumatically to the recipient site. If the fit is too tight, we might harm the periodontal ligament and thus cause harm to the donor tooth (Paulsen, Andreasen, and Schwartz 1995).

The use of a replica tooth could potentially help surgeons decrease the challenges associated with autotransplantation. Extra‐oral time and the degree of manipulation of the donor tooth are factors that could potentially be modified by using patient‐specific donor tooth replicas. Previously, it has been shown that premolars have a significantly more favorable prognosis than molars (Atala‐Acevedo et al. 2017; Jaber et al. 2024). This was also confirmed in the study of Juslin et al. (2020) that the 1‐year survival of transplanted premolars was 95% and 80% for molars. The prognosis of multi‐rooted teeth could potentially benefit from the use of replicas. Surgical success increases with increasing experience of the team of professionals (Juslin et al. 2020). A surgical guide could possibly assist novice surgeons in tooth transplantation and experienced surgeons in the challenging cases.

Concerning the autotransplantation of teeth with open apices, the time between CBCT scanning and the transplantation of the natural tooth should be as little as possible. This is crucial to ensure dimensional and contour equivalence of the natural tooth compared to the manufactured replica tooth. In a study of Verweij et al. they found out that prolonged extra alveolar time of over 3 min and multiple fitting attempts of the natural tooth were associated with low‐quality CBCT scans and a long interval between CBCT scanning and the autotransplantation procedure. It was also suggested in the study that CBCT scanning should be performed maximally 2 months before transplantation in the clinical setting (Verweij et al. 2020).

Sufficient space at the donor and recipient sites and favorable tooth morphology facilitates an atraumatic removal of the donor tooth. This study focused to modeling and reproduction of only the donor tooth. If appropriate software is available, it is also possible to create a preoperative virtual simulation of the whole surgical procedure and thus manufacture a separate guided template for drilling the alveolar bone (Shahbazian et al. 2010; Anssari Moin et al. 2016). In a case of several possible donor teeth, 3D technology can also be utilized in the decision making of the best possible donor for the selected recipient area (Kim, Choi, and Pang 2019).

Replica teeth can be fabricated with several CAM methods. The most used method is milling a model from biocompatible material. In this study, PEEK was chosen from many possible materials since its biocompatibility is well proven and its processability is good. It has been successfully used as an alloplastic biomaterial in craniofacial reconstructions (Scolozzi, Martinez, and Jaques 2007) as in orthopedic and spinal surgery (Kurtz and Devine 2007) for many years. Computer‐assisted 3D modeling can be used to manufacture medical applications from PEEK (Scolozzi, Martinez, and Jaques 2007) and the material can be sterilized by steam or gamma irradiation without changing the proportions of the model or causing degradation of the material (Shah, Jung, and Skirboll 2014). Dental laboratories can process data of scanned teeth in STL‐format and milling devices are frequently owned. The requirements of the European Medical Device Regulations (REGULATION (EU) 2017/745 OF THE EUROPEAN PARLIAMENT AND OF THE COUNCIL of 5 April 2017 on medical devices, amending Directive 2001/83/EC) for temporarily bone‐contacting biomaterials need to be considered when the system is used routinely and in commercial basis.

## 5 Conclusion

A CBCT scan maintains sufficient image quality for tooth autotransplantation planning. The replica tooth corresponded in size and shape to the natural tooth in terms of clinically expected need of precision.

## Author Contributions

Formulating the study question and hypothesis: Tuija Teerijoki‐Oksa, Päivi Jääsaari, Marja Ekholm, Pekka Vallittu, Lippo Lassila, Hanna Thorén. Desigining the study: Jessica Juslin, Tuija Teerijoki‐Oksa, Päivi Jääsaari, Marja Ekholm, Pekka Vallittu, Lippo Lassila, Hanna Thorén. Entering the data: Jessica Juslin. Drafting the manuscript: Jessica Juslin, Tuija Teerijoki‐Oksa, Päivi Jääsaari, Marja Ekholm, Pekka Vallittu, Lippo Lassila, Hanna Thorén. Validating the results: Tuija Teerijoki‐Oksa, Päivi Jääsaari, Marja Ekholm, Pekka Vallittu, Lippo Lassila, Hanna Thorén. Supervision: Tuija Teerijoki‐Oksa, Hanna Thorén.

## Conflicts of Interest

The authors declare no conflicts of interest.

## Data Availability

The data that support the findings of this study are available from the corresponding author upon reasonable request.
